# Platelet full length TFPI-α in healthy volunteers is not affected by sex or hormonal use

**DOI:** 10.1371/journal.pone.0168273

**Published:** 2017-02-03

**Authors:** Kristien Winckers, Stella Thomassen, Hugo ten Cate, Tilman M. Hackeng

**Affiliations:** 1 Department of Biochemistry, Cardiovascular Research Institute Maastricht (CARIM), Maastricht University, Maastricht, the Netherlands; 2 Department of Internal Medicine, CARIM, Maastricht University Medical Centre, Maastricht, the Netherlands; Institut d'Investigacions Biomediques de Barcelona, SPAIN

## Abstract

**Background:**

Only 10% of plasma TFPIα (TFPI) exists in the full length form, the rest circulates as a C-terminally truncated form. However, blood platelets exclusively contain full length TFPI, which is released at the site of injury upon platelet activation, and which could play an important local regulatory role in thrombin generation and prevention of thrombosis.

**Methods:**

The anticoagulant activities of full length and truncated TFPI were investigated using thrombin generation assays. Blood samples were obtained from 30 healthy volunteers (10 male subjects, 10 female subjects, and 10 females using oral contraceptives). Platelet TFPI was released in platelet rich plasma and in platelet isolates using convulxin or thrombin, and measured by free TFPI ELISA and thrombin generation assays.

**Results:**

Full length TFPI and platelet TFPI were much more potent inhibitors of thrombin generation than truncated TFPI, which was virtually inactive. Although mean plasma TFPI antigen levels decreased from men (0.30 nM) to women (0.20 nM) to women using oral contraceptives (0.11 nM), no relevant differences were found in platelet TFPI among those subgroups.

**Conclusions:**

Platelets release similar amounts of TFPI regardless of plasma TFPI concentrations and is unaffected by sex or oral contraceptive use. We speculate that platelet TFPI is important to prevent systemic coagulation and thrombosis and restrict thrombus formation to the site of the growing platelet plug. The stable contribution of platelet TFPI to the anticoagulant potential in plasma is likely to become particularly relevant under conditions of low plasma TFPI levels in combination of oral contraceptives use.

## Introduction

Tissue factor pathway inhibitor-ɑ (TFPI) is the main inhibitor of the extrinsic coagulation pathway and therefore an important modulator of coagulation initiation. TFPI consists of an acidic N-terminal region, three successive Kunitz domains and a basic C-terminal region [1–3]. The first and second Kunitz domains of TFPI are involved in the binding of TF/FVII(a) and FXa, respectively [1, 4]. For the rapid and efficient inhibition of FXa, the C-terminal region and all three Kunitz domains of TFPI are required [5, 6]. Moreover, the third Kunitz domain is responsible for the interaction of TFPI with protein S, which acts as a cofactor of TFPI by enhancing the inhibition of FXa ten-fold [3, 7, 8].

TFPI is mainly produced by the vascular endothelium. The majority (80%) of TFPI stays associated with the endothelium [9], whereas the remaining 20% of TFPI circulates in plasma at a concentration of approximately 2.5 nM. In plasma, only 20% of TFPI circulates as the free form and consists of either carboxy-terminal truncated TFPI (10%) or full length TFPI (10%) [10]. The remaining 80% of plasma TFPI is C-terminal truncated and bound to lipoproteins, predominantly to LDL [10]. Therefore, total TFPI and LDL covariate in plasma and as a result patients with abetalipoproteinemia, lacking apolipoprotein B, an important component of LDL, have very low levels of both LDL and total TFPI [11]. Remarkably, these patients do not suffer from a prothrombotic phenotype [11], indicating the relative unimportance of truncated plasma TFPI as a natural anticoagulant. Indeed in vitro experiments using diluted prothrombin based coagulation assays revealed a much stronger anticoagulant activity for full length TFPI compared to truncated and lipoprotein-associated forms of TFPI [6, 12]. The role of different truncated forms of TFPI is not yet known.

Platelets contain 5–10% of circulating TFPI [13–15] and in contrast to plasma TFPI, the platelet TFPI pool exclusively consists of full length TFPI. Therefore platelets embody almost half of circulating active full length TFPI. At the site of vascular injury, local TFPI concentrations might even increase through the release of TFPI from accumulating platelets within the thrombus. In clinical studies plasma TFPI rather than other sources of TFPI are investigated and therefore little is known about the importance and inter-individual variation of platelet versus plasma TFPI. In the present study we studied both recombinant and platelet derived full length TFPI and compared it with truncated TFPI forms. Moreover we investigated inter-individual functional differences in platelet TFPI and studied the influence of sex and the use of oral contraceptives on platelet versus plasma TFPI.

## Materials and methods

### Materials

Thrombin calibrator standard was obtained from Thrombinoscope BV (Maastricht, the Netherlands). Fluorogenic substrate I-1140 was obtained from BACHEM (Bubendorf, Switzerland). TF was supplied by Dade Innovin^®^ (Dade Behring, Marburg, Germany). Dioleoyl-sn-glycero-3-phosphocholine (DOPC), 1,2-dioleoyl-sn-glycero-3-phosphoserine (DOPS), and 1,2-dioleoyl-sn-glycero-3-phosphoethanolamine (DOPE) were obtained from Avanti Polar Lipids (Delfzijl, The Netherlands). Phospholipid vesicles (20% DOPS, 20% DOPE, and 60% DOPC) were prepared as described. Corn trypsin inhibitor (CTI) was from Haematologic Technologies Inc (Vermont, USA). Monoclonal antibodies against TFPI were purchased from Sanquin (Amsterdam, the Netherlands) and pooled together to prepare an anti-TFPI antibody cocktail consisting of 4 monoclonal antibodies directed against different epitopes of TFPI (clone CLB/TFPI Kunitz-1, Kunitz-2, Kunitz-3 and C-terminus). Truncated TFPI (TFPI_1-161_) and full length TFPI were kind gifts from Dr. Lindhout and Prof. Dr. Buurman from the Faculty of Health, Medicine and Life Sciences, respectively. Convulxin was obtained from Enzo Life Sciences BVBA (Antwerp, Belgium). Hirudin was obtained from Nodia BV (Antwerp, Belgium).

### Methods

#### Blood sampling

Blood was collected from healthy volunteers (18–55 years) from the antecubital fossa, using a 21 gauge needle. Tourniquet pressure was released during blood sampling and a discard tube was used before collecting citrate tubes (109 mmol/L trisodium citrate). Blood samples were processed immediately. Exclusion criteria were the use of acetylsalicylic acid or non-steroidal anti-inflammatory agents within one week prior to blood sampling and the use of anticoagulant drugs.

Subsequently, to investigate the effects of sex and oral contraceptive use on plasma and platelet TFPI, venous blood samples were obtained as described above from 30 healthy volunteers aged between 18 and 55 years old. Subjects were divided into 3 groups. The groups were 1) male subjects (n = 10), 2) female subjects not using oral contraceptives (OCs) (female subjects, n = 10) and 3) and female subjects using OCs (OC-users, n = 10).

Women have lower levels of plasma full length TFPI compared with men and in women the use of oral contraceptives is associated with a further reduction in full length TFPI by 25–45 [16–19]. The minimum sample size to detect a difference of 20% in platelet TFPI between at least two groups with a confidence level of 95% and a power of 0.80 is 9 for each group. We included ten individuals per group. This implies that given the current sample size differences of at least 20% will be detected. The study was approved by the Ethics Committee of Maastricht University Medical Center and conducted in accordance with the Declaration of Helsinki. All subjects gave written informed consent before participating.

### TFPI depleted plasma

For the preparation of TFPI depleted plasma a mixture of monoclonal anti-human TFPI antibodies was covalently coupled to cyanogen bromide-activated Sepharose according to manufacturer’s instructions. The coupled CNBr-activated Sepharose was washed 3 times with HN and blocked with HN containing 10% bovine serum albumin during 30 minutes. After an additional washing step, the Sepharose beads were incubated with pooled normal plasma in the presence of CTI for 60 minutes after which the beads were separated from the plasma by centrifugation. Remaining TFPI levels (< 1%) in TFPI-depleted plasma were measured with a full length TFPI ELISA as described [20].

### Plasma and platelet preparations

Platelet rich plasma (PRP) was prepared by centrifugation of citrated whole blood at 180 x g during 15 minutes without brake. Platelet poor plasma (PPP) was prepared by centrifugation of citrated whole blood at 2,000 x g during 15 minutes.

For the isolation of platelets, ACD-buffer (1:15 vol/vol) was added to the whole blood prior to centrifugation. Washed platelet isolates with a calculated platelet count of 500 x 10^9^/L were prepared from each donor essentially as described previously [21]. After centrifugation of 2,000 μL PRP containing ACD (1:15 vol/vol), the supernatants were discarded and the platelet pellets were resuspended in 1,000 μL platelet buffer (pH 6.6) containing ACD (1:15 vol/vol) and the platelets were spun down by centrifugation at 2,875 x g during 2 minutes. This washing step was repeated twice. Finally, the platelet pellets were resuspended in 200 μL platelet buffer (pH 7.5) and the platelet concentration was measured on a Microdiff 18 Coulter Counter (Beckman Coulter Nederland BV, Mijdrecht, The Netherlands). Raw platelet counts in blood and washed platelet preparations are shown (S1 Table).

### Platelet stimulation

Washed platelets were stimulated with thrombin (8 nM final concentration) or convulxin (50 ng/mL final concentration) and incubated during 10 minutes at 37°C after which the reaction of the thrombin activated platelets was stopped by the addition of hirudin (32 nM final concentration). The platelet supernatants were obtained by double centrifugation to remove any residual platelet fragments and stored at -80°C prior to analysis. First the platelet suspensions were centrifuged during 2 min at 2,875 x g and subsequently the suspensions were centrifuged during 10 minutes at 21,400 x g.

In a second experiment, PRP and PPP were prepared from each donor as described above. By adding autologous PPP, the platelet count of PRP was set at 250 x 10^9^/L. Corn trypsin inhibitor with a final concentration of 33 μg/mL was added to all samples (including the control sample) to prevent unwanted effects of contact activation induced by sample handling. The PRP samples were incubated with 50 ng/mL convulxin (final concentration) during 15 minutes at 37°C, after which the platelets were separated from the plasma by centrifugation at 11,000 x g during 10 minutes. As a control, to exclude release of TFPI solely due to plasma preparation, the PRP samples were incubated with HNBSA-buffer (vehicle) as well. The obtained PPP and PPP samples from stimulated (convulxin) and non-stimulated PRP (vehicle) were transferred into fresh tubes and stored at -80°C prior to analysis.

### Thrombin generation

Thrombin generation (TG) was measured by calibrated automated thrombin generation (CAT). Coagulation was initiated with 1–2 pM tissue factor as indicated in the figure legends, in the presence of 30 μM synthetic phospholipid vesicles and 16 mM CaCl_2_. The formation of thrombin in plasma was continuously monitored with a Fluoroscan Ascent reader (Thermo Labsystems, Helsinki, Finland) using the fluorogenic substrate I-1140. Each thrombin generation curve was calibrated against the fluorescence signal obtained in the same plasma sample with 100 nM thrombin calibrator using Thrombinoscope software (Thrombinoscope, Maastricht, the Netherlands). The inhibitory activity of increasing concentrations of recombinant full length TFPI and truncated (TFPI_1-161_) were studied in TFPI-deficient plasma by thrombin generation. In addition, the activity of platelet derived full length TFPI was studied in TFPI deficient plasma. For that purpose supernatants from convulxin-activated washed platelets isolates, (3*10^9^ plt/ml) containing released platelet TFPI, were used. TG experiments were performed in the presence of CTI (33 μg/mL) and both in the absence and presence of anti-TFPI antibodies. In a next experiment, TG was determined in PPP and in PPP from stimulated PRP in pooled plasma samples from male subjects, female subjects, and OC-users, both in the absence and in the presence of anti-TFPI antibodies.

### TFPI ELISA

Plasma and supernatant free TFPI antigen levels were quantified by ELISA (Asserachrom Free TFPI antigen assay, Diagnostica Stago, Asnieres, France). Data obtained from the commercial free TFPI ELISA kit were originally expressed as ng/mL but were converted to nanomolar (nM). For this purpose, a molecular weight of 43 kDa for the free, full length glycosylated TFPI molecule was used [3]. Raw data of TFPI measurements are shown (S1 Table).

### Data handling

Data are presented as mean ± standard deviation, unless indicated otherwise. Differences in plasma or supernatant TFPI antigen level before and after platelet stimulation were analyzed using a paired t-test. Differences in supernatant or plasma TFPI levels between subgroups were tested using analysis of variance (ANOVA). Post hoc analysis was performed using the Bonferroni or Games-Howell post hoc test, as appropriate. Statistical analysis was performed using SPSS 22.0.

## Results

**Fig 1:**
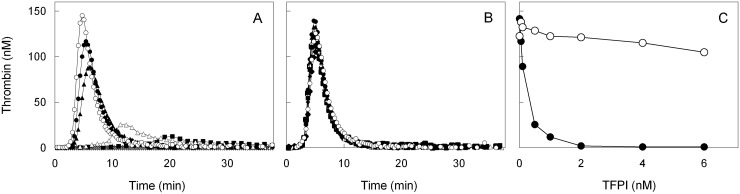
Effects of C-terminal truncated and full length TFPI on the regulation of thrombin generation. Thrombin generation by 2 pM of tissue factor in TFPI deficient plasma reconstituted with increasing concentrations of A: full length TFPI or B: C-terminal truncated TFPI. Thrombin generation is shown at final concentrations of 0 nM TFPI (○); 0.05 nM (●); 0.1 nM (▲); 0.5 nM (△); 1 nM (■): 2 nM (□); and 4 nM (◆) and 6 nM (▯). C: Thrombin peak heights from panel A and B are shown as a function of concentration of full length TFPI (●) or C-terminal truncated TFPI (○). Averages of duplicates are shown.

The anticoagulant activity of full length vs C-terminal truncated TFPIα (TFPI) was measured in thrombin generation assays in TFPI deficient plasma to which increasing concentrations of either truncated TFPI or full length TFPI were added (Fig 1). The addition of full length TFPI potently inhibited TG, which was completely blocked at concentrations of TFPI > 1 nM (Fig 1A–1C), while the addition of truncated TFPI up to 4 nM did not affect thrombin generation (Fig 1B and 1C). To illustrate the activity of platelet derived full length TFPI, supernatants from convulxin stimulated washed platelets from healthy volunteers were added to TFPI-deficient plasma after which thrombin generation was measured both in the presence and absence of anti-TFPI antibodies.

**Fig 2:**
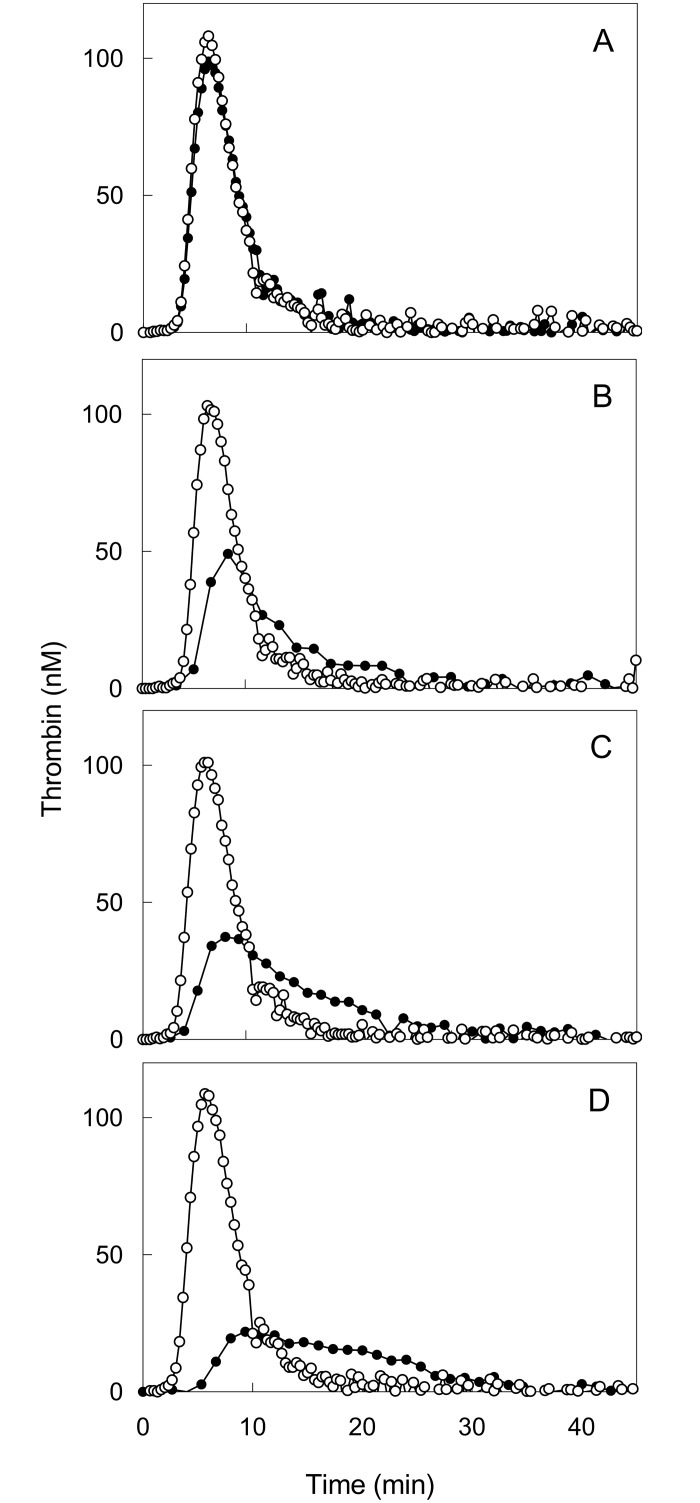
Anticoagulant effect of recombinant and platetet derived TFPIα on thrombin generation. Thrombin generation was measured at 1 pM tissue factor in the presence of 30 μM phospholipid vesicles and 16 mM CaCl_2_ in the absence (●) and presence (○) of anti-TFPI antibodies in TFPI deficient plasma (panel A-D). The TFPI deficient plasma is supplemented with buffer (panel A), reconstituted with 0.20 nM final concentration recombinant full length TFPI (panel B), and supernatant from convulxin activated washed platelet isolates (panel C; D) resulting in 0.26 nM (panel C) and 0.49 nM (panel D) final concentration full length TFPI.

The addition of TFPI neutralizing antibodies did not affect thrombin generation measured in TFPI-deficient plasma reconstituted with buffer, indicating that no relevant amounts of TFPI remained in the TFPI-depleted plasma (Fig 2A). The addition of full length TFPI (0.2 nM final concentration) inhibited thrombin generation peak height by approximately 50% (Fig 2B). Addition of neutralizing anti-TFPI antibodies completely restored thrombin generation to the situation in absence of TFPI (Fig 2A and 2B). Similar inhibition of TG was achieved upon the addition of a supernatant derived from convulxin-activated washed platelets obtained from two healthy volunteers. The final TFPI concentrations in plasma from these supernatants were 0.26 nM (Fig 2C) and 0.49 nM (Fig 2D), respectively. Under these conditions thrombin generation was decreased dose-dependently, beyond the effect of 0.2 nM recombinant TFPI, suggesting that platelet TFPI has a comparable anticoagulant activity to recombinant TFPI. In either case, addition of neutralizing anti-TFPI antibodies completely restored thrombin generation, indicating that the anticoagulant effect was exclusively due to platelet TFPI present in the supernatant (Fig 2C and 2D).

**Fig 3:**
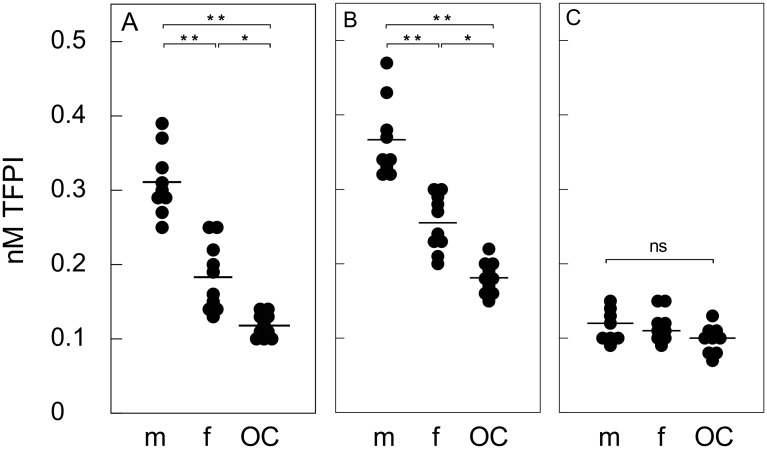
Plasma and platelet TFPI levels in males, female, and oral contraceptive users. Individual TFPI levels (ng/ml) in healthy male subjects (m) female subjects (f) and oral contraceptive users (OC) are shown in PPP (panel A) and PPP from activated PRP (250 x 109 platelets/L) stimulated by convulxin (50 ng/mL) (panel B). TFPI release from each individual is shown. Mean released TFPI levels in platelet supernatants adjusted to a platelet count of 250 x 109/L are shown (Panel C). Averages of duplicates and mean TFPI levels are shown. * P-value < 0.01, ** P-value< 0.001.

To study the influence of sex and the use of oral contraceptives on platelet TFPI, thirty healthy volunteers with a mean age of 28 were included in the study (range 21–45 years). Mean age was comparable between groups (male subjects: 28.4 ± 1.9 years, female subjects: 30.2 ± 2.4 years and OC-users: 26.7 ± 1.0, p-value 0.429) and mean whole blood platelet count was 257 ± 50 x 10^9^/L. (range 21–45 years). Baseline mean TFPI antigen level antigen level in plasma was 0.20 ± 0.09 nM. Data on TFPI from one male subject were excluded from analysis because this subject was identified as an extreme outlier (0.84 nM TFPI). As expected, plasma TFPI antigen levels differed between the subgroups (Table 1), with the highest levels observed in male subjects (0.31 ± 0.05 nM TFPI) followed by female subjects (0.18 ± 0.05 nM TFPI) and OC-users (0.12 ± 0.02 nM TFPI) (Fig 3A). Upon convulxin-induced platelet stimulation, mean overall TFPI antigen levels measured in plasma increased to 0.27 ± 0.09 nM TFPI (p-value < 0.001). For the different subgroups, plasma TFPI antigen levels measured upon platelet stimulation varied between 0.37 nM, 0.26 nM, and 0.18 nM TFPI for male subjects, female subjects, and OC users, respectively (Fig 3B; Table 1). Remarkably, in all cases the calculated amount of TFPI released by activated platelets was similar at 0.06 nM (Table 1).

**Table 1:** Platelet TFPIα in healthy volunteers.

	TFPIα in PPP (nM)	TFPIα in PPP^Ŧ^ (nM)	TFPIα release (nM)	TFPIα release washed platelets (nM)^†^
males (n = 9)	0.31 ± 0.05	0.37 ± 0.03	0.06 ± 0.03 ^ns^	0.12 ± 0.02 ^ns^
females (n = 10)	0.18 ± 0.05**	0.26 ± 0.04**	0.07 ± 0.03 ^ns^	0.11 ± 0.02 ^ns^
OC-users (n = 10)	0.12 ± 0.02*	0.18 ± 0.02*	0.06 ± 0.03 ^ns^	0.10 ± 0.02 ^ns^

Mean released TFPI levels in platelet poor plasma (PPP), and in platelet poor plasma from activated platelet rich plasma (PPP^Ŧ^) in healthy male subjects, female subjects, and oral contraceptive (OC) users. Calculated TFPI release is shown as well as the TFPI release in platelet supernatants of washed platelets adjusted to a platelet count of 250 x 10^9^/L.

** P-value < 0.001 female vs male subjects,

* P-value < 0.01 OC-users vs female subjects;

ns: no significant differences between all subgroups. ^†^ Data on TFPI release from washed platelets are excluded from one male subject, one female subject and one oral contraceptive user as their platelet count in isolated decreased by more than 20% during sample preparation, indicating spontaneous platelet activation.

Compared with the baseline PPP sample (0.20 ± 0.09 nM TFPI), TFPI levels were also slightly higher in the PPP sample obtained from PRP treated with vehicle (free TFPI 0.21 ± 0.09 nM, p-value < 0.05). This slight increase probably reflects TFPI release as a result of partial platelet stimulation due to sample handling.

Washed platelet isolates were prepared from each individual. In all subjects there was a decrease in platelet count during the preparation of washed platelets isolates of at least 10%. In three subjects the decrease was more than 20% and those samples were excluded from analysis as the decrease might result from spontaneous platelet activation. Mean released TFPI levels in platelet supernatant adjusted to a platelet count of 250 x 10^9^/L were 0.11 ± 0.02 nM. Although OC-users has slightly lower levels of platelet derived TFPI, no significant differences were observed in released platelet TFPI measured in the supernatants among subgroups (male subjects: 0.12 ± 0.02 nM, female subjects: 0.11 ± 0.02 nM and OC-users: 0.10 ± 0.02 nM, p-value = 0.104) (Fig 3C; Table 1).

**Fig 4:**
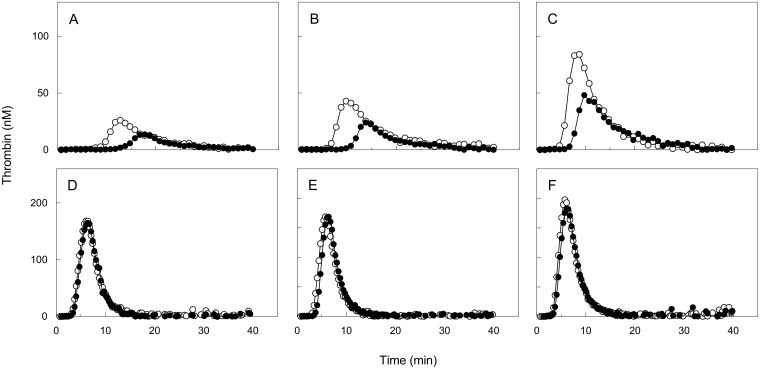
Contribution of platelet TFPI to anticoagulant potential in males, females, and oral contaceptive users. Thrombin generation measured at 1 pM tissue factor in the presence of 30 μM phospholipid vesicles and 16 mM CaCl_2_ in pooled PPP (○) and pooled PPP from convulxin activated PRP (●) in male subjects (A) female subjects (B) and OC-users (C). Comparative thrombin generation measured at 1 pM tissue factor in pooled PPP (○) and pooled PPP from convulxin activated PRP (●) in male subjects (D) female subjects (E) and OC-users (F) in the presence of neutralizing anti-TFPI antibodies. PPP was derived from PRP (250 x 109 platelets / L) stimulated by convulxin (50 ng/mL)

Thrombin generation assays in PPP and in PPP from activated PRP were performed to determine whether the released TFPI was functionally active. The peak height and endogenous thrombin potential (ETP) measured at 1 pM TF in PPP was lowest in pooled plasma from male subjects (Fig 4A), followed by female subjects (Fig 4B) and OC-users (Fig 4C). Peak heights were 26 nM, 43 nM and 84 nM in normal PPP and 14 nM, 24 nM and 49 nM thrombin in PPP from stimulated PRP for male subjects, female subjects, and OC-users, respectively, reflecting a considerable anticoagulant effect of TFPI released by platelets. In the presence of neutralizing antibodies against TFPI no differences were observed in peak heights measured in PPP and PPP from stimulated PRP (Fig 4D–4F). Peak heights were 169 nM vs. 165 nM in male subjects (Fig 4D), 168 nM vs. 169 nM in female subjects (Fig 4E), and 197 nM vs. 184 nM thrombin in OC-users (Fig 4F), for PPP and PPP from stimulated PRP in the presence of anti-TFPI antibodies, respectively. This indicates that under current experimental conditions TFPI is the major determinant of TG (Fig 4).

## Discussion

In the current study we have shown that only full length TFPI is able to efficiently inhibit thrombin formation in plasma in contrast to its C-terminal truncated forms that represent the majority of TFPI in plasma.

Although platelets only contain 5–10% of circulating TFPI, the fact that they exclusively contain full length TFPI suggests that platelet derived TFPI represents a potent resource of inhibition of thrombin generation. To examine this, TFPI-deficient plasma was reconstituted with supernatants of convulxin-stimulated washed platelets obtained from healthy volunteers. Platelets contain a myriad of proteins (amongst which factor V), which are released upon their activation [22, 23]. Nonetheless, the net effect of the addition of these platelet supernatants on thrombin generation was inhibitory and could be completely neutralized by anti-TFPI antibodies, illustrating that platelet derived TFPI within the supernatant is responsible for the down-regulation of thrombin generation in plasma. Recently, it was shown that TFPI from platelets was able to inhibit FXa [24], it is currently shown that platelet TFPI can also effectively regulate thrombin generation.

It is known that TFPI is an essential protein, as no TFPI deficient individuals are known, and TFPI null mice die before birth [25]. However, no clear association between low plasma TFPI level and risk on (venous) thrombosis has been observed [17, 26–29]. If TF is truly important in the pathogenesis of thrombosis [30], variations in TFPI would have a clear impact on thrombus formation, unless plasma TFPI does not represent the functional TFPI pool. First evidence for a biological role of platelet TFPI was observed in a murine model of FVIII deficiency in which the function of platelet TFPI was examined [31]. Hemophiliac mice lacking hematopoietic TFPI showed a decrease in blood loss upon tail transection compared with hemophiliac mice with intact platelet TFPI. Moreover, the lack of platelet TFPI was associated with increased fibrin depositions following femoral vein injury [31]. TFPI is involved in coagulation initiation, and therefore it is conceivable that platelet TFPI modulates bleeding as well as thrombotic disorders.

The study of platelet TFPI in humans is inconvenient and therefore little functional studies on human platelet TFPI have been performed so far. In the present study the functional variation in plasma and platelet TFPI in 30 healthy volunteers was investigated. In line with previous observations, plasma TFPI was the lowest in women using OCs, followed by women not using OCs and men [18, 19, 32]. It is thought that these low TFPI levels explain at least partially the increased thrombotic risk associated with OC use [33]. The release of platelet TFPI was investigated both in plasma environment and in washed platelet isolates. Notably, the amount of platelet TFPI was not relevantly affected by either sex or the use of OCs. When corrected for a platelet count of 250 x 10^9^/L, platelet supernatant TFPI for all groups was 0.11 ± 0.02 nM. Based on these data 250 x 10^9^ / L platelets contain on average 62 ± 26% of plasma free TFPI. As plasma TFPI has a large inter individual variation, the relative contribution of platelet TFPI differs greatly between subgroups (male subjects: 38 ± 7%, female subjects: 65 ± 25% and OC-users 81 ± 22%). The amount of TFPI released in plasma environment was half of the amount of TFPI released in washed platelet isolates. This might partly be explained by the use of different platelet agonists (convulxin versus thrombin). To investigate if platelet TFPI had additional functional activity in the presence of plasma TFPI, TG was studied in PPP and in PPP obtained from stimulated PRP, which revealed an average 43% reduction in peak height in the presence of platelet derived TFPI. In the presence of anti-TFPI antibodies the effects of platelet stimulation on thrombin generation disappeared, again supporting the hypothesis that the inhibitory effects can be contributed to TFPI originating from the stimulated platelets.

In search of the roles of different forms of TFPI in regulation of coagulation it is now established that truncated TFPI does not function as an important anticoagulant in plasma and that platelet TFPI forms a potent TFPI storage pool. During their activation platelets expose phosphatidylserine and provide an efficient catalytic surface for the assembly of the tenase and prothrombinase complexes enhancing thrombin generation [23]. By transforming into these procoagulant surfaces platelets contribute to the thrombus architecture and prevent systemic coagulation. It is tempting to speculate that platelets regulate local thrombus formation at the site of vascular injury also through the release of TFPI, especially under conditions where plasma TFPI is low due to for example OC-use. As TFPI concentrations locally increase, an anticoagulant environment is created surrounding the injury, in which free FXa and membrane-bound TF/FVIIa are scavenged from the circulation in order to prevent unwanted thrombus formation. Nonetheless, clinical studies focusing on different pools of TFPI and their anticoagulant contributions are needed to unravel the role of different forms and locations of TFPI.

## Supporting information

S1 Table Raw data platelet count and platelet TFPI measurements. Group number indicates male subjects (group 1), female subjects (group 2), OC-users (group 3) (I). Platelet count was measured in blood (II), and in platelet isolates (VII). PRP was adjusted with PPP to 250*10^3^/μL. Free TFPI was measured in PPP (III), in PPP derived from PRP incubated with convulxin BSA (IV) and in PPP derived from PRP incubated with BSA (V). * indicates omitted data (see manuscript). TFPI released by platelets in plasma was calculated (VI). TFPI was also measured in supernatants obtained from washed platelets (VII) incubated with convulxin (VIII), and recalculated to 250*10^3^/μL platelet count (IX).
(PDF)
