# Supplementary material for: Platelet full length TFPI-α in healthy volunteers is not affected by sex or hormonal use
Source: PLoS One. 2017 Feb 3;12(2):e0168273. doi: 10.1371/journal.pone.0168273 (PMC5291377; doi:10.1371/journal.pone.0168273)
Supplement: S1 Table — Group number indicates male subjects (group 1), female subjects (group 2), OC-users (group 3) (I). Platelet count was measured in blood (II), and in platelet isolates (VII). PRP was adjusted with PPP to 250*103/μL. Free TFPI was measured in PPP (III), in PPP derived from PRP incubated with convulxin BSA (IV) and in PPP derived from PRP incubated with BSA (V). * indicates omitted data (see manuscript). TFPI released by platelets in plasma was calculated (VI). TFPI was also measured in supernatants obtained from washed platelets (VII) incubated with convulxin (VIII), and recalculated to 250*103/μL platelet count (IX). (PDF) [file pone.0168273.s001.pdf]

S1 File: Raw data platelet count and platelet TFPI measurements.

I	II	III	IV	V	VI	VII	VIII	IX
group	**platelet count** in whole blood (*10^3/μL)	**free TFPI (nM)** in PPP from whole blood	**free TFPI (nM)** free TFPI PRP to PPP CVX	**free TFPI (nM)** in PRP to PPP BSA	**free TFPI (nM)** Calculated (IV-III)	**platelet count** in platelet isolate (*10^3/μL)	**free TFPI (nM)** from washed platelets CVX uncorrected	**free TFPI (nM)** from washed platelets CVX corrected (250 *10^3/μL)
**Male**								
1a	448	0.29	0.34	0.27	0.05	448	0.22	0.12
1b	398	0.33	0.33	0.3	0.00	398*	0.19*	
1c	446	0.84*	0.82*	0.81*		446	0.24	0.13
1e	441	0.39	0.43	0.37	0.04	441	0.24	0.14
1f	454	0.25	0.32	0.3	0.07	454	0.19	0.10
1g	446	0.3	0.38	0.34	0.08	446	0.27	0.15
1h	454	0.31	0.37	0.35	0.06	454	0.17	0.09
1i	436	0.37	0.47	0.41	0.10	436	0.24	0.14
1j	457	0.27	0.32	0.26	0.05	457	0.16	0.09
1k	465	0.29	0.34	0.3	0.05	465	0.18	0.10
**average:**	**445**	**0.31**	**0.37**	**0.32**	**0.06**	**450**	**0.21**	**0.12**
**Female**								
2a	450	0.14	0.23	0.15	0.09	450	0.26	0.14
2b	457	0.13	0.20	0.13	0.07	457	0.27	0.15
2c	452	0.20	0.30	0.25	0.10	452	0.18	0.10
2e	455	0.22	0.30	0.24	0.08	455	0.16	0.09
2f	447	0.25	0.28	0.25	0.03	447	0.21	0.12
2g	251	0.14	0.23	0.15	0.09	251*	0.09*	
2h	450	0.25	0.27	0.24	0.02	450	0.2	0.11
2i	419	0.16	0.24	0.16	0.08	419	0.19	0.11
2j	454	0.19	0.29	0.21	0.10	454	0.21	0.12
2k	447	0.15	0.21	0.16	0.06	447	0.17	0.10
**average:**	**428**	**0.18**	**0.26**	**0.19**	**0.07**	**448**	**0.21**	**0.11**
**OC-user**								
3a	450	0.13	0.22	0.13	0.09	450	0.19	0.11
3b	439	0.11	0.17	0.15	0.06	439	0.22	0.13
3c	441	0.12	0.2	0.15	0.08	441	0.19	0.11
3e	466	0.1	0.15	0.1	0.05	466	0.19	0.10
3f	431	0.11	0.16	0.1	0.05	431	0.17	0.10
3g	438	0.13	0.18	0.11	0.05	438	0.12	0.07
3h	468	0.14	0.2	0.15	0.06	468	0.15	0.08
3i	448	0.1	0.19	0.09	0.09	448	0.18	0.10
3j	398	0.1	0.18	0.1	0.08	398*	0.14*	
3k	444	0.14	0.16	0.14	0.02	444	0.15	0.08
	**442**	**0.12**	**0.18**	**0.12**	**0.06**	**447**	**0.17**	**0.10**

Group number indicates male subjects (group 1), female subjects (group 2), OC-users (group 3) (I). Platelet count was measured in blood (II), and in platelet isolates (VII). PRP was adjusted with PPP to 250*10^3/μL. Free TFPI was measured in PPP (III), in PPP derived from PRP incubated with convulxin BSA (IV) and in PPP derived from PRP incubated with BSA (V). * indicates omitted data (see manuscript). TFPI released by platelets in plasma was calculated (VI). TFPI was also measured in supernatants obtained from washed platelets (VII) incubated with convulxin (VIII), and recalculated to 250*10^3/μL platelet count (IX).
